# Rare Presentation of Bilateral Lobular Capillary Hemangioma of the Nasal Septum: A Case Report

**DOI:** 10.7759/cureus.31262

**Published:** 2022-11-08

**Authors:** Abdullah Alabdulqader, Yazeed Alsuliman

**Affiliations:** 1 Otolaryngology - Head and Neck Surgery, College of Medicine, Imam Mohammad Ibn Saud Islamic University (IMSIU), Riyadh, SAU; 2 Otolaryngology - Head and Neck Surgery, King Fahad Medical City, Riyadh, SAU

**Keywords:** benign nasal mass, nasal hemangioma, nasal septum, lobular capillary hemangioma, bilateral

## Abstract

Lobular capillary hemangioma (LCH) is a benign, rapidly growing lesion affecting the skin and mucous membranes. LCH is common in the oral cavity but is rarely observed in the nasal cavity. The disease etiology is not fully understood; however, trauma and hormonal changes are attributable factors. Patients usually present with nasal obstruction and epistaxis. This study reports the clinical picture, diagnosis, and management of a rare case of bilateral septal nasal LCH.

## Introduction

Lobular capillary hemangioma (LCH) is a benign, rapidly growing lesion that affects the skin and mucous membranes of the oral and nasal cavities [1]. However, the disease etiology is incompletely understood. The most common site of involvement is the oral cavity [2-3]. Most sinonasal cases of LCH are unilateral at presentation. This study describes a rare case of bilateral nasal septal LCH that was successfully treated using transnasal endoscopic excision.

## Case presentation

**Figure 1:**
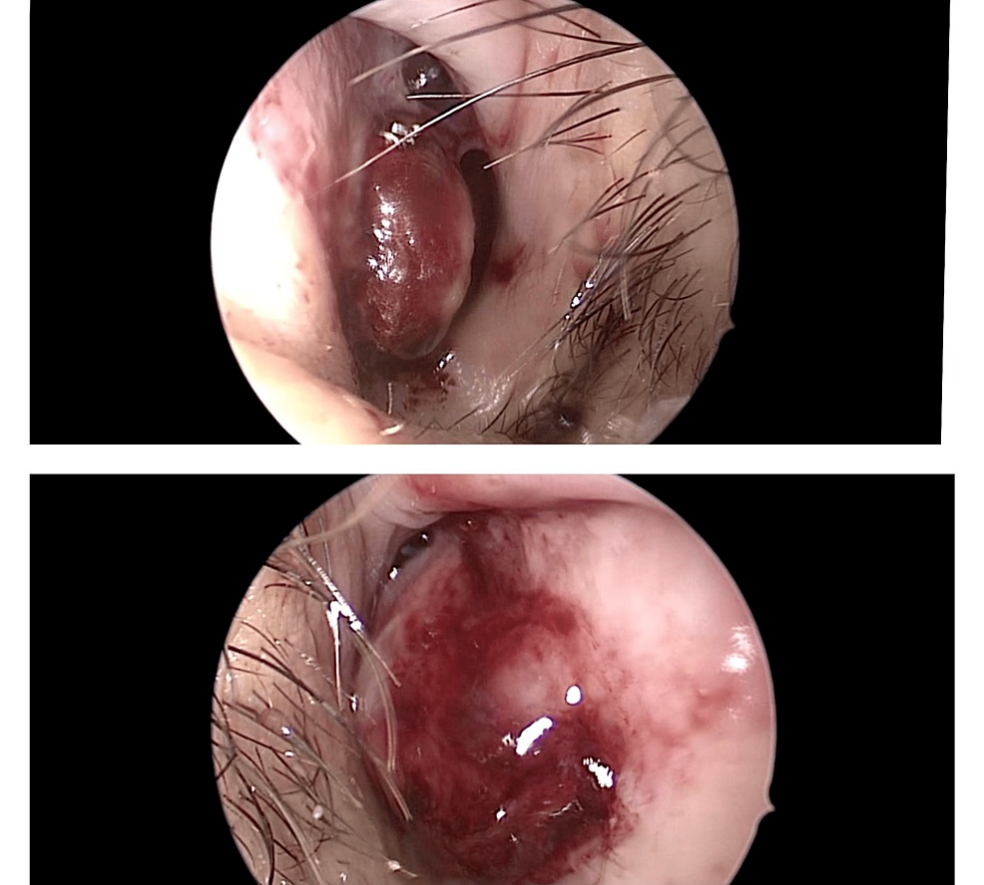
Endoscopic examination shows a bilateral reddish hemorrhagic polypoid lesion in the nasal septum.

**Figure 2:**
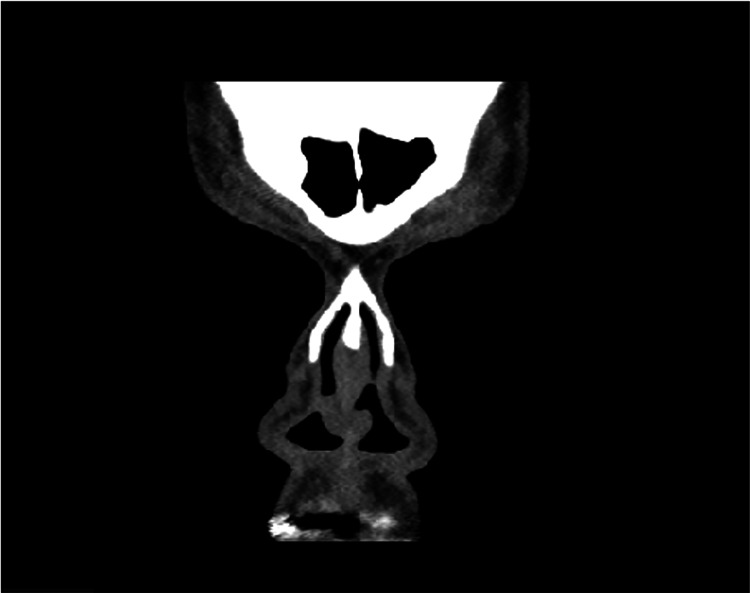
A computed tomography scan of nasal and paranasal sinuses shows an anterior cartilaginous nasal septal lesion.

A 49-year-old female patient was referred from a secondary hospital for additional examinations and management. She had hypertension and hypothyroidism and presented with complaints of multiple episodes of bilateral epistaxis, nasal obstruction, postnasal drip, and facial pain. These symptoms persisted for nine months. She denied a history of allergies, recent infections, trauma, or chronic rhinosinusitis. She underwent right nasal mass excision and right-sided limited functional endoscopic sinus surgery (FESS) at the referring hospital. The biopsy results suggested LCH. Symptoms recurred a few months later, with reduced quality of life and depression. Clinical evaluation included nasal endoscopic examination, which revealed a bilateral reddish hemorrhagic polypoid lesion (approximately 1.0 × 1.0 cm) in the caudal region of the nasal septum bilaterally and opposing. The lesion occluded both nostrils and bled on touch (Figure 1). Sinonasal examination revealed healthy mucosa and a clear ostiomeatal complex. The results of ear, throat, and head and neck examinations were unremarkable. Contrast-enhanced computed tomography of the nasal cavity and paranasal sinuses showed a bilateral anterior cartilaginous nasal septal lesion (1.2 × 0.7 × 1.0 cm) (Figure 2). No underlying bony erosion or significant nasal septal deviation was observed. There was mild right maxillary sinus mucosal thickening after FESS.

The patient was prepared for endoscopic nasal mass excision under general anesthesia. Intraoperatively, a Killian incision was made caudal to the lesion on both sides. The mucoperichondrial flap was elevated until it reached the posterior, superior, and inferior margins of the lesion. There was iatrogenic septal cartilage perforation. Thus, the bilateral presentation in our patient may be due to the migration of a previous lesion through the perforation. The lesion was excised with the removal of the surrounding mucoperichondrium. To cover the bare cartilage area, an anteriorly based septal mucosal flap was harvested posterior to the lesion and rotated to cover the left side of the septum, where mucosal loss was relatively large. Silastic sheets were used.

The postoperative course was uneventful, and the patient received oral antibiotics for 10 days, with a follow-up of 10 days. At follow-up, the silastic sheets were removed, and nasal endoscopic examination revealed bilateral caudal crusting adherent to the septum. Partial debridement was performed, and the nasal mucosa healed properly. The final diagnosis was LCH.

## Discussion

Lobular capillary hemangiomas are benign lesions of the skin and mucous membranes. This type of hemangioma is also known as pyogenic granuloma, which is a misnomer because the lesions are neither contagious nor granulomatous. The lesions can be pedunculated or sessile and range from a few millimeters to several centimeters [3]. LCH is usually unilateral, and its etiology is unknown [4-5]. However, pathogenesis is related to trauma, hormonal effects, viral oncogenes, microscopic arteriovenous malformations, and the production of angiogenic growth factors [6]. LCH is more common in women. Nevertheless, this entity can occur in males and individuals of all ages [4]. The most affected sites include the gingiva, lips, tongue, and oral mucosa. The nasal cavity is affected in a few cases, and the most commonly affected regions of the nasal cavity are the anterior septal mucosa and the tip of the turbinate [1-7]. The typical presentation is nasal obstruction, epistaxis, epiphora, and purulent rhinorrhea [7]. Treatment involves local surgical excision, and recurrence can be avoided by exposing the cartilage and strip of the underlying mucoperichondrium [3]. Recurrence is rare, and lesions are benign [8]. All reported sinonasal LCH cases are unilateral at presentation. In our case, the biopsy showed bilateral caudal septal LCH. The bilateral presentation in our case may be due to the migration of a previous lesion through an iatrogenic septal cartilage perforation.

## Conclusions

LCH is a rare disease of unknown etiology. Pathogenesis may be related to hormonal, traumatic, viral, oncogenic, and other factors. LCH should be suspected when a vascular lesion is diagnosed in the nasal cavity. The treatment of choice is the surgical excision of the cuff of the surrounding mucoperichondrium. The bilateral presentation of LCH may be due to the migration of a lesion through an iatrogenic septal perforation in previously operated patients with residual lesions or recurrence. Although rare, LCH should be differentiated from a bleeding mass in the nasal cavity.
